# A joint analysis using exome and transcriptome data identifiescandidate polymorphisms and genes involved with umbilical hernia in pigs

**DOI:** 10.1186/s12864-021-08138-4

**Published:** 2021-11-13

**Authors:** Igor Ricardo Savoldi, Adriana Mércia Guaratini Ibelli, Maurício Egídio Cantão, Jane de Oliveira Peixoto, Michele Porto Pires, Marcos Antônio Zanella Mores, Essamai Brizola Lagos, Jader Silva Lopes, Ricardo Zanella, Mônica Corrêa Ledur

**Affiliations:** 1 Programa de Pós-Graduação em Zootecnia, Universidade do Estado de Santa Catarina, UDESC-Oeste, Chapecó, SC 89815-630 Brazil; 2 Embrapa Suínos e Aves, 321, Concórdia, SC 89715-899 Brazil; 3 Programa de Pós-Graduação em Ciências Veterinárias, Universidade Estadual do Centro-Oeste, Guarapuava, PR 85040-167 Brazil; 4 Instituto Catarinense de Sanidade Agropecuária, Florianópolis, SC 88034001 Brazil; 5 Programa de Pós-Graduação em Zootecnia, Universidade Estadual de Ponta Grossa, Ponta Grossa, PR Brazil 84030-900; 6 BRF S.A, Curitiba, Brazil PR 82305-100; 7 Universidade de Passo Fundo, Passo Fundo, RS 99052-900 Brazil; 8 Programa de Mestrado em BioExperimentação, Universidade de Passo Fundo, Passo Fundo, RS 99052-900 Brazil

**Keywords:** Candidate genes, Congenital defects, RNA-Seq, SNP, Swine

## Abstract

**Background:**

Umbilical Hernia (UH) is characterized by the passage of part of the intestine through the umbilical canal forming the herniary sac. There are several potential causes that can lead to the umbilical hernia such as bacterial infections, management conditions and genetic factors. Since the genetic components involved with UH are poorly understood, this study aimed to identify polymorphisms and genes associated with the manifestation of umbilical hernia in pigs using exome and transcriptome sequencing in a case and control design.

**Results:**

In the exome sequencing, 119 variants located in 58 genes were identified differing between normal and UH-affected pigs, and in the umbilical ring transcriptome, 46 variants were identified, located in 27 genes. Comparing the two methodologies, we obtained 34 concordant variants between the exome and transcriptome analyses, which were located in 17 genes, distributed in 64 biological processes (BP). Among the BP involved with UH it is possible to highlight cell adhesion, cell junction regulation, embryonic morphogenesis, ion transport, muscle contraction, within others.

**Conclusions:**

We have generated the first exome sequencing related to normal and umbilical hernia-affected pigs, which allowed us to identify several variants possibly involved with this disorder. Many of those variants present in the DNA were confirmed with the RNA-Seq results. The combination of both exome and transcriptome sequencing approaches allowed us to better understand the complex molecular mechanisms underlying UH in pigs and possibly in other mammals, including humans. Some variants found in genes and other regulatory regions are highlighted as strong candidates to the development of UH in pigs and should be further investigated.

**Supplementary Information:**

The online version contains supplementary material available at 10.1186/s12864-021-08138-4.

## Background

Umbilical hernia (UH) is a condition that negatively affects pigs, being considered the most common congenital defect in this species [1]. In addition to economic losses caused by reduced performance, UH results in welfare concerns to the modern pig industry [1–3]. This condition occurs due to weakened support of muscles around the umbilical ring or umbilicus of the animal [4], causing the non-closing of the umbilical area properly. In consequence, the intestines protrude through the abdominal wall to form the herniary sac [4]. Moreover, the involvement of collagen production and metabolism in hernia development was previously identified as described in a recent review by Nowacka-Woszuk [5].

The etiology of UH is likely to be multifactorial, affected by genetic and environmental factors, such as physical injury, obesity, inappropriate removal of the umbilical cord and infections [6, 7]. It has been reported that the occurrence of UH ranges from 0.40 to 2.25%, affecting mainly 9 to 14 week-old pigs [1, 8]. Moreover, animals under the same management conditions may be affected or not by hernias [8]. The heritability estimates of 0.06 to 0.08 [8, 9] for UH in pigs indicate that the development of this condition is partially controlled by a genetic component.

Few studies have already been developed seeking to understand the UH genetic inheritance. Ding et al. [9] observed significant linkage between markers and scrotal/inguinal and umbilical hernia in pigs on 12 different chromosomes. Liao et al. [10] identified two suggestive loci predisposing to umbilical hernia on SSC2 and SSC17 in a Duroc population. In a genome-wide association study (GWAS) with commercial pigs, Fernandes at al [11]. identified five SNPs associated with umbilical hernia: one in SSC4 (rs334706328), two in SSC6 (rs80813241, rs81337222), one in SSC13 (rs337360700) and the other with unknown position in the pig genome. Moreover, Grindflek et al. [12] studying the Norwegian Landrace pigs, identified a highly significant Quantitative Trait Loci (QTL) for umbilical hernia, detected between 48 and 51 Mb on SSC14.

Even though some studies have been performed, they suggested that this disorder is complex and affected by multiple genes and causal variants. Thus, further studies are required to identify additional susceptibility loci and causative genes for UH in pigs using different strategies. Therefore, to clarify the genetic basis of swine umbilical hernia, this study aimed to identify polymorphisms and genes associated with UH in pigs through the whole-exomic sequencing and additional transcriptome data analyses.

## Methods

### Animals and sample collection

A total of 10 unrelated Landrace purebred females (with approximately 90 days of age) was used in a case-control design. These gilts were selected from the same nucleus farm with high sanitary status, located in Santa Catarina State, south of Brazil. From those, 5 were affected by umbilical hernia and 5 were healthy selected from families with no history of any type of hernia. For each case, a contemporary and unrelated control animal was used. The animals were transported to the Embrapa Swine and Poultry National Research Center to be necropsied and to confirm the presence or absence of UH, as described by Souza et al. [13]. The euthanasia was performed by electrocution for 10 s following the procedure approved by the Embrapa Swine and Poultry National Research Center Ethical Committee of Animal Use (CEUA) under the protocol number 011/2014. For the exome analysis, samples from the ear tissue were collected and stored at − 20 °C until DNA extraction. For the transcriptome analysis, samples were collected from the umbilical ring tissue, immediately frozen in liquid nitrogen and stored at − 80 °C until RNA extraction.

### DNA isolation

Genomic DNA was extracted from 70 to 100 mg of ear tissue using Purelink Genomic DNA Mini kit (Thermo Fisher Scientific, Waltham, MA, USA). Briefly, tissue digestion was performed adding 200 μL of Genomic Digestion Solution Buffer and 20 μL Proteinase K for 4 h at 55 °C. The samples were centrifuged at 14,000 rpm for three minutes at room temperature, and 20 μL RNAse, 200 μL Pure Link Lysis/Binding Buffer and 200 μL 100% alcohol were added. The solution was pipetted into the silica column with the washing steps performed with 500 μL Wash Buffer 1 and 2 centrifuged at 12000 rpm per 1 min to bind DNA to the silica column. Finally, the DNA was eluted in 50 μL Elution Buffer solution. The concentration and quality of samples were measured in a Biodrop spectrophotometer (Biodrop, England, UK) and in a 1,5% agarose gel electrophoresis. Only DNA samples showing the 260/280 ratio between 1.8 and 2.0 were used for further analyses.

### Exomic capture and sequencing

The exon capture indexing was performed separately for each sample. To prepare the next generation sequencing (NGS) libraries, the SeqCap EZ Exome Probes v1.0 kit (Roche NimbleGen, Madison, WI, USA), which was designed in the Sscrofa 10.2 genome, was used. The DNA fragmentation was performed using the Bioruptor® equipment (Diagenode, Denville, NJ, USA) following the recommendations of the protocols. The gDNA was fragmented to an insertion size of approximately 150 bp, generating dsDNA fragments with 3′ or 5′ overhangs to index the transport adapters for sequencing. Samples with a concentration of 10 ng/μL diluted in TE (10 mM Tris, 1 mM EDTA, pH 7.5–8.0) were pipetted into 0.1 mL Bioruptor Microtubes. The sonication was performed in three stops of seven minutes each containing 15 cycles (30″/ 30″ on / off time) submerged in water at 4 °C.

After fragmentation, the clean-up of fragmented DNA was performed using purification beads (SPB), followed by the blunt end repair (ERP3). After the final repair, the library size was selected using SPB beads. Next, the adapters were ligated and samples were prepared for the probe hybridization. Finally, the enrichment of the DNA fragments was performed, as this process selects and enriches the DNA fragments that have adapters at the ends and amplifies the amount of DNA in the library. Furthermore, sequencing was performed in Illumina’s HiSeq 2500 at the ESALQ/USP Functional Genomics Center (São Paulo/Brazil) using a paired-end 100 bp library.

### Exome sequencing analysis and annotation

The FASTQ files were submitted to quality control (QC) analysis using the Trimmomatic tool [14] to remove low-quality sequences (PHRED ≤20). The remaining reads were mapped against the *Sus scrofa* reference genome (Sscrofa11.1) using the BWA-MEM [15]. All the SNPs were analyzed and identified individually for each sample. Variant calling (SNP and InDel) was performed with GATK tool v.3.6 following the general guidelines for whole-exome sequencing (WES) [16]. The variant effect predictor (VEP) tool available in the Ensembl 103 [17] was used to annotate and identify the effects and consequences of all variants that differed between normal and UH-affected pigs. For this analysis, the data resulting from the GATK were saved in the VCF format. The list of variants was submitted to the VEP tool from Ensembl 103 using its standard criteria, in which additional identifiers for genes (gene symbol, transcript version, and protein), transcripts and variants were used (transcript biotype, exon and intron numbers, phenotypes and Upstream/Downstream distance 5000 bp), co-located variants and frequency data.

Additionally, the sorting intolerant from tolerant (SIFT) score [17, 18], available in the VEP tool, was used to identify the potential impact of amino acid substitutions on protein structure and function, which can, consequently, alter the phenotype. The use of this tool implies in a better prediction of the effect of non-synonymous coding variants [19]. The SIFT score is given for each variant by which one can predict whether the variant can affect the protein function or not. A SIFT score ranging from 0.00 to 0.05 classifies a variant as deleterious and from > 0.05 to 1.00 as tolerated.

### Transcriptome variants analysis

The variant calling of the umbilical ring tissue transcriptome between healthy and herniated pigs were performed with samples from the Bioproject PRJNA445856. The RNA from these samples was extracted with a combined protocol using Trizol Reagent and Qiagen RNeasy mini kit, and the library preparation was performed using the TruSeq mRNA Stranded Sample Preparation kit (Illumina Inc., San Diego, CA, USA). Libraries were sequenced in the same lane, in a paired end protocol (2x100bp) in Illumina’s HiSeq 2500 at the ESALQ/USP Functional Genomics Center (São Paulo/Brazil). This transcriptomic data was previously described by Souza et al. [13], which was generated with the same animals used for the exome sequencing. The sequences generated in the transcriptome analysis were submitted to quality control using the Trimmomatic tool [14] to remove low-quality sequences (PHRED ≤20) and mapped against the reference genome *Sus scrofa* (NCBI *Sus scrofa* 11.1) using the STAR tool [20]. The identification of different variants between both groups was performed using the GATK tool v. 3.6, following the Guide of Best Practices for using GATK [16]. After mapping, the Picard tool (https://broadinstitute.github.io/picard/) was used to add the sort, read groups and marking duplicates parameters. In the GATK, the same parameters were used in the exome and RNA-Seq analysis, except for the split’N’Trim (to split “N” cigar reads), which was used only in the RNA-Seq dataset. The mapping qualities were reassigned, bases recalibrated for each sample and the variant detection was performed using GATK HaplotypeCaller. To filter low quality variants, the following parameters were used: SNP cluster considering a window 35:3, FS > 30.0, QD < 5.0, MQ < 50.0, MQRankSum < − 12.5, ReadPosRankSum < − 8.0 and GQ < 5.0. To select the variants, a QUAL ≥30.0 and DP ≥ 100.0 was used. Subsequently, the data obtained was submitted to the VEP Ensembl 103 program for annotation and prediction of variants using the same input criteria as those used for the exome analysis. Afterwards, the concordance of variants found with both WES and RNA-Seq approaches were verified.

### Gene ontology and functional analysis

To evaluate the functions of the genes identified in common with the exome and transcriptome approaches, the DAVID 6.8 (https://david.ncifcrf.gov) [21] and Panther databases (http://www.pantherdb.org/) [22] were used to classify the gene ontology (GO) categories of cellular component, biological process (BP) and molecular function using swine and human information. Afterwards, the BP enriched with genes through the DAVID were grouped using REViGO (http://revigo.irb.hr) [23] for better visualization. Interactions between genes were predicted with the NetworkAnalyst program (https://www.networkanalyst.ca) using the information available on human annotation [24]. Furthermore, it was verified whether the genes found in our study were in QTL regions previously mapped for umbilical hernia in pigs or not using the Pig QTLdb from the Animal Genome Database (http://www.animalgenome.org/QTLdb/app) [25].

### Polymorphism validation

Five variants identified in our study were chosen to be validated with Sanger methodology.

Primers were designed using Primer-Blast program with sequences downloaded from the Ensembl databases (Table 1). PCR reactions contained final volume of 20 μL, with 1X reaction buffer, 2.0 mM of MgCl2, 0.4 mM of dNTPs, 0.2 μM of each primer, 0.2 U of Go Taq polymerase (Promega, Madison, WI, USA) and 30.0 ng of genomic DNA were prepared. The PCR was performed under the following conditions: denaturing at 94 °C for 2 min, followed by 35 cycles of denaturing at 95 °C for 30 s, annealing at 56 or 57 °C °C for 30s, extension at 72 °C for 30 s and final extension for 5 min at 72 °C. PCR bands were confirmed by electrophoresis and then, sequencing reactions were prepared using the BigDye® Terminator v3.1 Cycle Sequencing Kit (Applied Biosystems, Foster City, CA, USA) using forward and reverse primers. Sequencing was performed in ABI 3130xl Genetic Analyzer (Applied Biosystems, Foster City, CA). Sequences were analyzed in the PhredPhrapConsed and those regions with Phred quality > 20 were considered for polymorphism identification.

**Table 1:** Primers used for variants validation with Sanger

Gene/ Ensembl ID	Sequence (5′- > 3′)	Annealing temperature
ELOA/	F: CACGGAATCTAAAGCCACAG	57 ° C
ENSSSCG00000025440	R: ACTAGAGGCCAAAGCCAA	
MYH8/	F: CTGCCCAAGGTCATACATAC	57 ° C
ENSSSCG00000018005	R: GGAATCTCCGCAGTAAAGC	
ZNF629/	F: CTGTGTGTGGTGTAATCCTC	56 ° C
ENSSSCG00000007780	R: ACTCAGGTAGTGGAATCAGG	
ITGAM/	F: GTGCTGGGTTAGGGTGAAT	56 ° C
ENSSSCG00000007754	R: GGGAAAGGAGTGAGGAGGA	
CCT6A/	F: AGCATTCATGCCTGTCTTGG	56 °C
ENSSSCG00000033141	R: AACCTTGGGTCGGGTTGATT	

## Results

### Whole-exomic analysis

Approximately 260 million of paired end reads were obtained for all samples, with an average of 25.9 million per sample. After the quality control, 94.61% of the reads were kept, with about 24.6 million paired end reads per sample (Table 2).

**Table 2:** Reads number per samples and reads kept after quality control

Samples	Input Read Pairs	Read Pairs Kept after QC
HE20	26,116,483	24,802,328
HE21	25,967,577	24,686,193
HE26	29,533,490	28,103,911
HE27	26,768,883	25,249,912
HE28	30,997,747	29,512,340
HE29	25,636,430	24,213,740
HE30	17,397,892	16,274,415
HE31	22,101,532	20,574,080
HE36	28,354,266	26,937,402
HE37	26,791,299	25,551,296

**Fig. 1:**
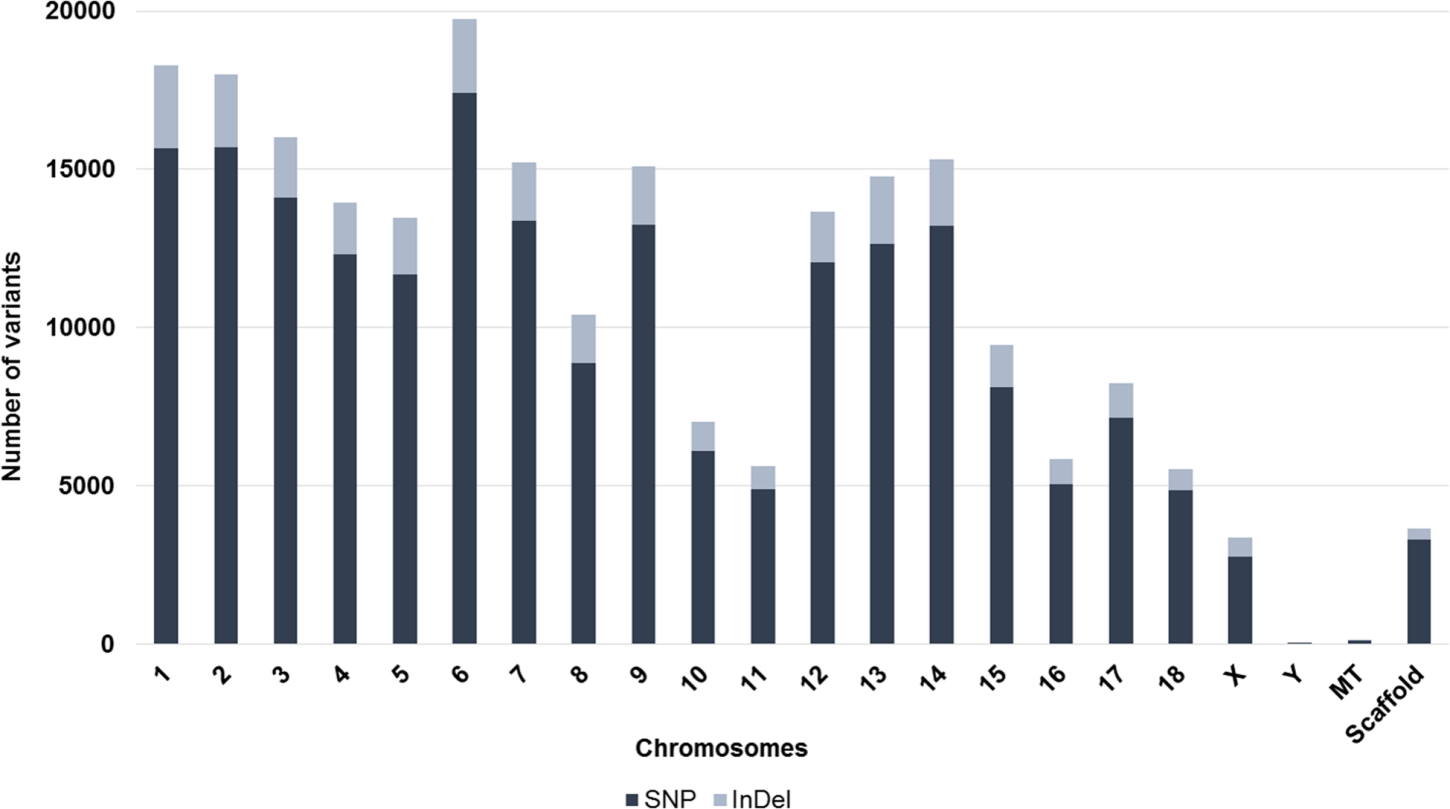
Total number of variants detected in each chromosome using the pig exome sequencing

Using the GATK for variant discovery, a total of 232,808 variants (SNPs and InDels) were identified in all 10 samples evaluated. The highest number of variants was found in the swine chromosome (SSC) 6, 1 and 2, respectively (Fig. 1).

**Fig. 2:**
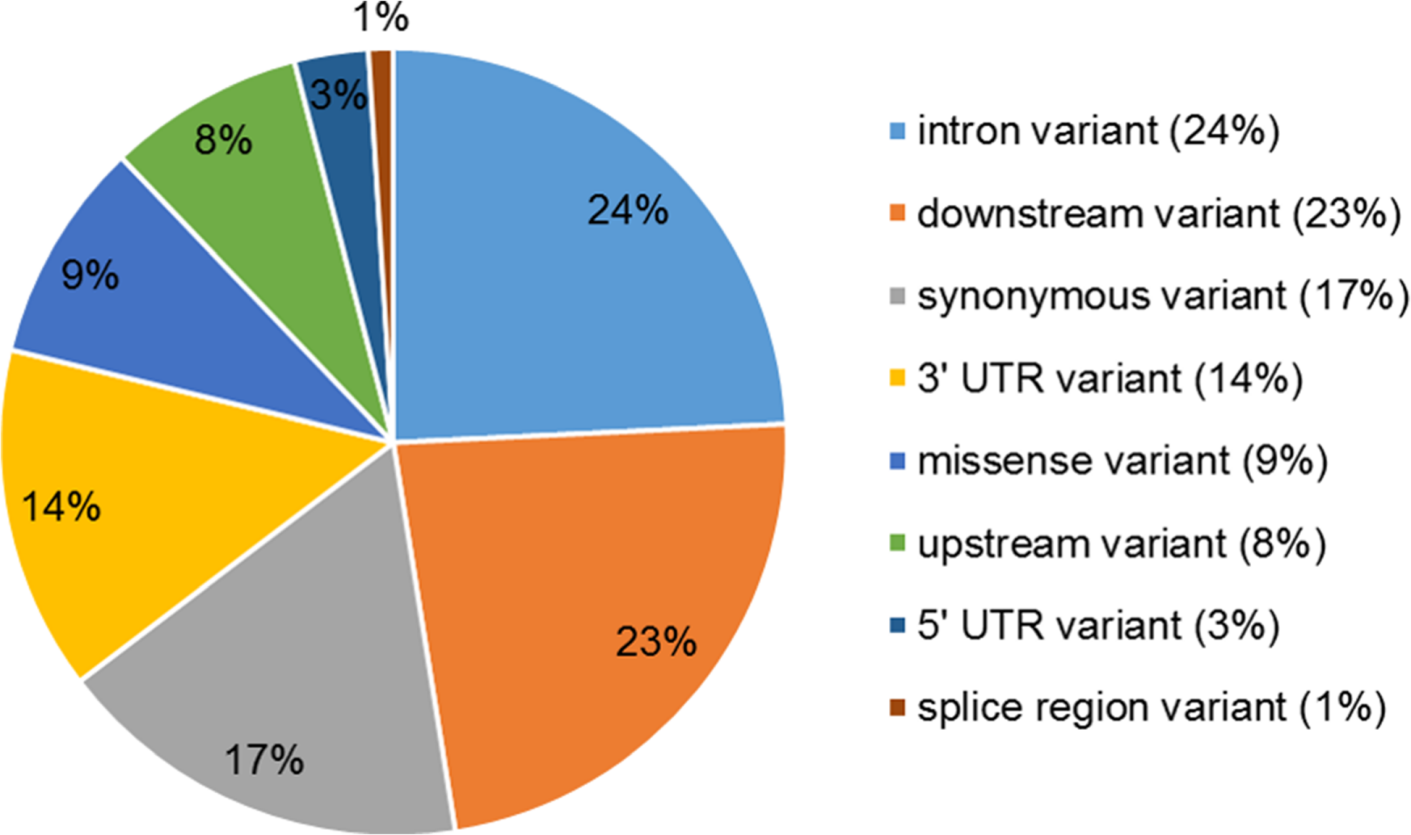
Classification of 119 variants (SNPs and InDels) generated with the VEP tool when analyzing the pig exomic sequences according to their position in genes

Considering only the variants between the two groups (healthy and UH-affected pigs), 119 polymorphisms were identified, where 9 were Indels and 110 SNPs (Additional file 1: Table S1). From those, 94 have already been described, and the other 25 are new polymorphisms that were firstly described here. From the 119 variants, using the VEP tool, 1 was classified in an intergenic region and 118 were classified in gene regions (Fig. 2) according to their coding consequence, where 66% were grouped as synonymous and 34% as missense variants.

All the variants that differed between healthy and UH-affected pigs were located in the autosomes, with the largest number mapped in the SSC 3, 6 and 12 (Additional file 1: Table S1). The SSC3 had the largest number of variants identified, with a total of 74 variants located in 26 genes. On SSC 6, 18 variants were identified, located in 7 genes and on SSC12, 12 variants were identified in 11 genes (Additional file 1: Table S2)*.*

From those 119 total variants, 1 was classified in a chromosome scaffold (Additional file 1: Table S1) and 9 were insertion and deletion (InDels) located in 15 genes. The other 106 variants were classified as SNP and were located in several genes (Additional file 1: Table S2).

Variants were located in introns, upstream, downstream and also in spliced regions (Fig. 2). Considering only the coding consequences, 67% were synonymous and 33% were missense. Some of the missense variants in the *MYH8*, *MYH4* and *ENSSSCG00000036685* were firstly identified in this study (Table 3).

**Table 3:** Variants obtained with whole-exome sequencing located in 58 genes in the swine genome

Variant position	Gene symbol
Exon	*MYO19, MYH8, MYH3, ENSSSCG00000038539, MYH4, ENSSSCG00000007733, ENSSSCG00000033141, CCT6A, ZNF713, ENSSSCG00000036685, ITAGM, ZNF646, SETD1A, STX4, RNF40, HSD3B7, CCDC189, TAMALIN, EPHB2, ELOA* and *OTOP1.*
Intron	*DHRS11, MYH13, MYH8, MYH3, MYH4, UNC5D, SLC22A11, ENSSSCG00000033141, RARB, ENSSSCG00000047703, MMS19, UNC5D, SLC22A11, KCTD7, ENSSSCG00000007733, ZNF713, SEPTIN14, CCT6A, SETDA1, ZNF629, RNF40, CCDC189, DEDD, NIT1, TAMALIN, EPHB2, TEX46, LUZP1, LYPLA2, CNR2, FUCA1, PAPLAN, PDLIM5* and *RAPGEF5.*
Upstream	*MYH3, HSD3B7, SETD1A, ORAI3, RNF40, PFDN2, CNR2, CLIC4, UBASH3B, LOC100514433, ENSSSCG00000049720, LOC106509673, ENSSSCG00000018553, ENSSSCG00000033141, SUMF2* and *ENSSSCG00000007735*
5′ UTR	*ZNF713, BCKDK, ZNF629, RNF40, CNR2, ENSSSCG00000036685* and *ENSSSCG00000007733.*
Downstream	*DHRS11, ADPRM, KCTD7, ZNF713, PRSS53, VKORC1, HSD3B7, STX1B, SETD1A, RNF40, ZNF629, PHKG2, ROCK2, SLC66A3, NIT1, EPHB2, ENSSSCG00000047341, ENSSSCG00000018553, ENSSSCG00000033141* and *ENSSSCG00000027233*
3′ UTR	*ZNHIT3, MYO19, DHRS11, KCTD7, CCT6A, ZNF713, STX1B, HSD3B7, ORAI3, ZNF629, PHKG2* and *EPHB2.*

The 119 polymorphisms (Additional File 1: Table S1) identified in the whole exome sequencing between healthy and UH-affected pigs were located in 58 different Ensembl gene IDs with 47 annotated genes (Table 2). However, several polymorphisms were located in the same gene, for example, the *KCTD7* gene had 10 polymorphisms, most of which were located downstream and 3 ‘UTR. The *ZNF713* gene had 19 variants, some of which located in 3’ UTR, and the *EPHB2* gene had 13 polymorphisms located in different gene positions (Additional File 1: Table S2).

### Variants in the transcriptome analysis

**Fig. 3:**
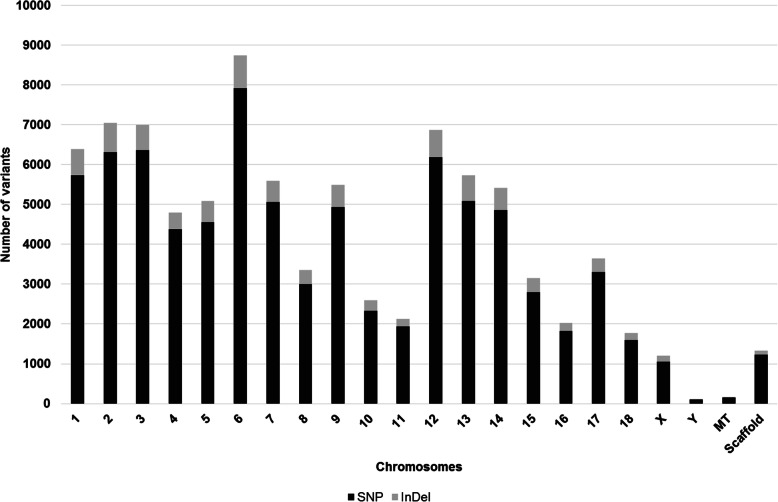
Total number of variants detected in each chromosome using the transcriptome sequences from normal and umbilical hernia-affected pigs

When analyzing variants in the umbilical ring transcriptome of the same 10 samples, a total of 89,638 polymorphisms (SNP and InDel) were identified. The highest number of variants was found in chromosomes 6, 2, 3 and 12, respectively (Fig. 3).

**Fig. 4:**
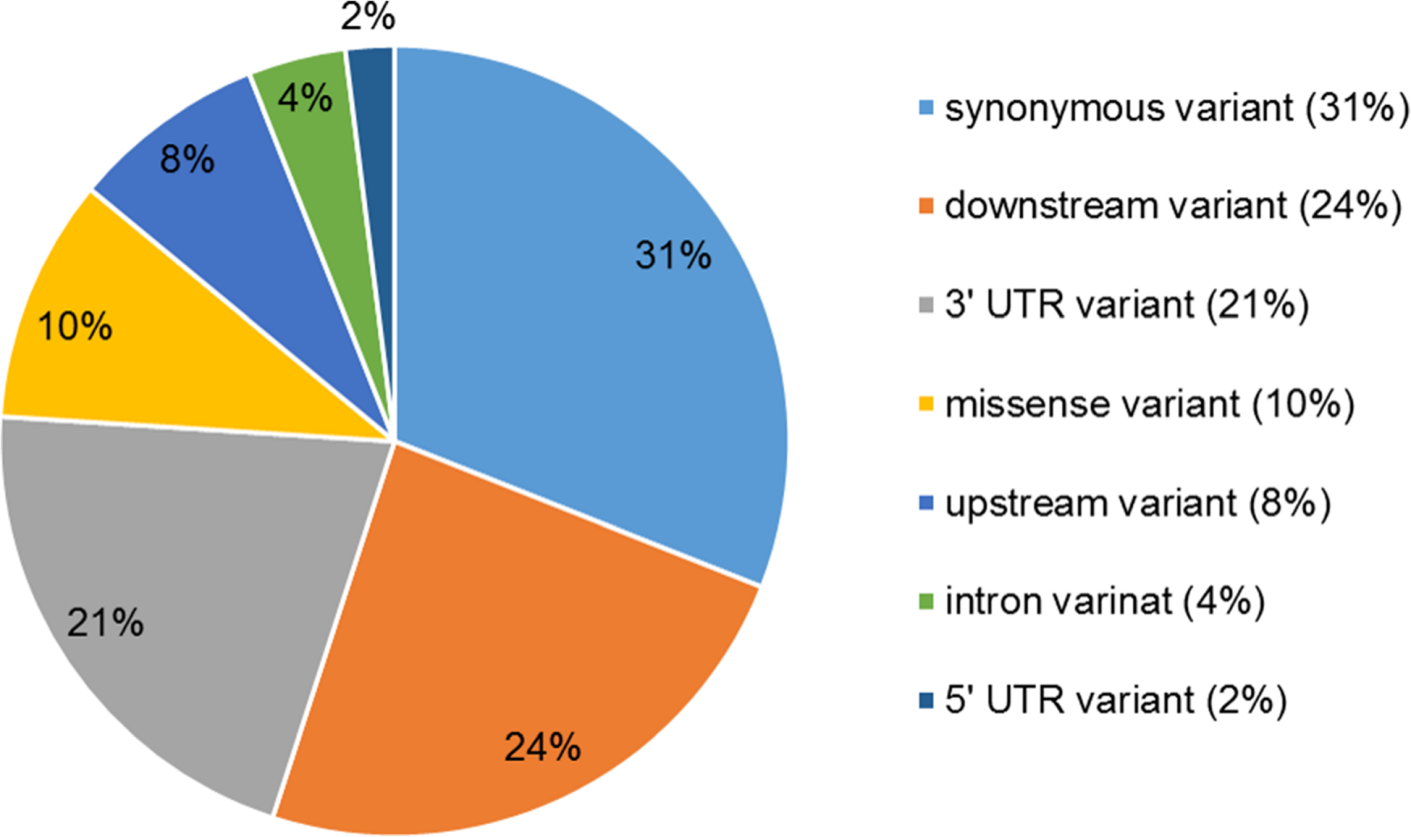
Classification of 46 variants (SNPs and InDels) generated with the VEP tool when analyzing the pig transcriptomic sequences according to their position in genes

To identify possible polymorphisms related to UH, the comparison between the two groups resulted in 46 different polymorphisms (SNPs and InDels) between herniated and non-herniated pigs (Additional file 1: Table S1). The 46 variants were located in coding regions, introns and flanking or regulatory regions. From those, 42 (91.3%) were existing variants and 4 (8.7%) were new, and they were classified according to their coding consequence, where 76% were synonymous and 24% missense variants (Additional file 1: Table S3). Only 3 variants were InDels, located in the *CCT6A, CLIC4, ZNF629*, *EOGT* and *RNF40* gene regions*.* Furthermore, similar to the exome results, some variants were classified according to the gene position in more than one gene, where 16 (41%) variants were located in exons (comprising 10 synonymous, 6 missense variants), 5 (4%) in introns and 48 (55%) in flanking regions (including 16 (24%) downstream, 9 (8%) upstream, 4 (2%) 5′ UTR and 19 (21%) 3’UTR variants) (Fig. 4).

The 46 polymorphisms between normal and UH-affected pigs were located in 27 genes (Table 4). Some genes harbored a high number of variants, such as the *KCTD7* gene, with 5 variants located in the 3 ‘UTR and downstream positions, whereas the *EPHB2* gene had 6 variants located in exons (synonyms and missense variants) and 3’ UTR, and the *ITGAM* gene presented 3 exon variants (synonyms and missense variants).

**Table 4:** Variants obtained with transcriptome sequencing located in the 27 genes in the swine genome

Variant position	Gene symbol
Exon	*ACACA, ENSSSCG00000007733, CCT6A, ZNF713, ITGAM, ZNF646, SETD1A, RNF40, ORAI3, ZNF629, EPHB2* and *ELOA.*
Intron	*DHRS11, KCTD7, SLC5A2, ZNF629* and *CCT6A*
Downstream	*DHRS11, CREB3L2, KCTD7, PRSS53, VKORC1, SETD1A, STX1B, RNF40, ENSSSCG00000033141* and *ENSSSCG00000018553*
Upstream	*HSD3B7, SETD1A, SUMF2, ENSSSCG00000033141* and *ENSSSCG00000018553*
3′ UTR	*DHRS11, EOGT, CREB3L2, KCTD7, CCT6A, HSD3B7, RUSF1, ORAI3, ZNF629, EPHB2* and *CLIC4.*
5′ UTR	*ZNF713, BCKDK* and *ZNF629*

The genotypes for the normal and UH-affected pigs for each polymorphism found between groups based on WES and RNA-Seq are shown in Additional file 1: Table S1.

### Variants with predicted effects on proteins

The VEP tool in the Ensembl 103 uses the extension SIFT which is a value that predicts whether an amino acid substitution affects the function of the protein or not. The general status of the SIFT score obtained using VEP is summarized in Table 5.

**Table 5:** Missense variants observed between normal and umbilical-hernia affected pigs with SIFT score calculated in the dbSNP database (Ensembl)

Method	Existing variation	Location	Gene Symbol	Exon Number	SIFT
Exomic	rs335136145	12:38026003–38,026,003	*MYO19*	21/26	tolerated (0.36)
	rs335136145	12:38026003–38,026,003	*MYO19*	21/27	tolerated (0.36)
	rs335136145	12:38026003–38,026,003	*MYO19*	18/23	tolerated (0.32)
	new	12:55146905–55,146,905	*MYH8*	23/41	tolerated (0.53)
	new	12:55146905–55,146,905	*MYH8*	22/40	tolerated (0.52)
	new	12:55146905–55,146,905	*MYH8*	24/42	tolerated (0.49)
	new	12:55267170–55,267,170	*MHY2*	20/40	tolerated (1)
	new	12:55267170–55,267,170	*MHY2*	19/39	tolerated (1)
	new	12:55267170–55,267,170	*MHY2*	21/41	tolerated (1)
	rs337918521	3:16960969–16,960,969	*ZNF713*	6/6	deleterious (0.02)
	rs323115420	3:16964045–16,964,045	*ZNF713*	5/6	tolerated (0.65)
	new	3:17033960–17,033,960	*ENSSSCG00000036685*	1/1	tolerated low confidence (0.07)
	new	3:17034293–17,034,293	*ENSSSCG00000036685*	1/1	tolerated low confidence (1)
	rs327289001	3:17254444–17,254,444	*ITGAM*	3/30	deleterious (0.01)
	rs327289001	3:17254444–17,254,444	*ITGAM*	2/30	deleterious (0.01)
	rs327289001	3:17254444–17,254,444	*ITGAM*	3/31	deleterious (0.01)
	rs327289001	3:17254444–17,254,444	*ITGAM*	2/31	deleterious (0.01)
	rs327289001	3:17254444–17,254,444	*ITGAM*	3/32	deleterious (0.01)
	rs337670844	3:17399477–17,399,477	*ZNF646*	3/4	tolerated (0.11)
	rs337670844	3:17399477–17,399,477	*ZNF646*	2/4	tolerated (0.1)
	rs337670844	3:17399477–17,399,477	*ZNF646*	1/2	tolerated (0.08)
	rs330957838	3:17468302–17,468,302	*SETD1A*	16/21	tolerated low confidence (0.34)
	rs789266896	3:17628688–17,628,688	*RNF40*	15/22	tolerated (0.62)
	rs789266896	3:17628688–17,628,688	*RNF40*	14/21	tolerated (0.62)
	rs789266896	3:17628688–17,628,688	*RNF40*	13/20	tolerated (0.6)
	rs325089032	6:81571496–81,571,496	*ELOA*	2/11	tolerated (0.1)
	rs325089032	6:81571496–81,571,496	*ELOA*	2/12	tolerated (0.1)
	rs696812713	8:6157581–6,157,581	*OTOP1*	7/8	tolerated (0.58)
	rs712855168	8:6157582–6,157,582	*OTOP1*	7/8	tolerated (0.53)
Transcriptomic	rs325089032	6:81571496–81,571,496	*ELOA*	2/11	tolerated (0.1)
	rs325089032	6:81571496–81,571,496	*ELOA*	2/12	tolerated (0.1)
	rs327289001	3:17254444–17,254,444	*ITGAM*	3/30	deleterious (0.01)
	rs327289001	3:17254444–17,254,444	*ITGAM*	2/30	deleterious (0.01)
	rs327289001	3:17254444–17,254,444	*ITGAM*	3/31	deleterious (0.01)
	rs327289001	3:17254444–17,254,444	*ITGAM*	2/31	deleterious (0.01)
	rs327289001	3:17254444–17,254,444	*ITGAM*	3/32	deleterious (0.01)
	rs789266896	3:17628688–17,628,688	*RNF40*	15/22	tolerated (0.62)
	rs789266896	3:17628688–17,628,688	*RNF40*	14/21	tolerated (0.62)
	rs789266896	3:17628688–17,628,688	*RNF40*	13/20	tolerated (0.6)
	rs330957838	3:17468302–17,468,302	*SETD1A*	16/21	tolerated low confidence (0.34)
	rs337670844	3:17399477–17,399,477	*ZNF646*	3/4	tolerated (0.11)
	rs337670844	3:17399477–17,399,477	*ZNF646*	2/4	tolerated (0.1)
	rs337670844	3:17399477–17,399,477	*ZNF646*	1/2	tolerated (0.08)
	rs323115420	3:16964045–16,964,045	*ZNF713*	5/6	tolerated (0.65)

From the exome analysis, according to the SIFT prediction, 14 variants of the SNP type were classified as missense and 2 variants were classified as deleterious in the *ZNF713* and in the *ITGAM* genes with SIFT score of 0.02 and 0.01, respectively. Moreover, 10 SNPs were designated as tolerated with the SIFT score ranging from 0.10 to 1.00 and 3 SNPs were classified as tolerated with low confidence on the SET domain containing 1A, histone lysine methyltransferase (*SETD1A*) and on the *ENSSSCG00000036685* (LOC17033960 and LOC17034293) genes with SIFT scores ranging from 0.07 to 1.00 (Table 5).

When analyzing the RNA-Seq variants*,* 7 were classified as missense on chromosomes 3 and 6, located in 7 genes. There was only one deleterious variant according to the SIFT score located in the *ITGAM* gene and one classified as tolerated with low confidence in the *SETD1A* gene. The other polymorphisms were classified as tolerated and were located in the *ZNF713*, zinc finger protein 646 (*ZNF646*), *RNF40* and *ELOA* genes (Table 5).

### Polymorphisms and common genes identified with the exome and transcriptome approaches

A total of 34 identical variants were found with both methodologies (Additional file 1; Table S4). From these, 4 variants were located in 2 genes of the zinc finger protein family *(ZNF629* and *ZNF713),* 9 variants in 4 genes of anti-inflammatory response and immune system *(ITGAM, RNF40, PRSS53* and *DHRS11),* 2 in the *SETD1A* gene, 3 variants in 2 genes of amino acid metabolism *(ACACA* and *HSD3B7),* 8 variants in 2 genes of blood pressure and size *(CCT6A* and *EPHB2),* 1 polymorphism in the *ELOA* gene*,* 1 variant in the *STX1B,* 4 polymorphisms in 2 genes of calcium and potassium modulator *(ORAI3* and *KCTD7),* and 2 variants in 2 new genes *ENSSSCG00000007733* and *ENSSSCG00000033141* (Additional File 1: Table S4)*.* Comparing the exome with the umbilical ring transcriptome [13], 36 genes were unique in the exome, 5 genes were unique in the transcriptome and 22 genes were common to both approaches. The common genes between the exome and transcriptome analysis were: *EPHB2, SUMF2, ITGAM, PRSS53, ORAI3, ZNF629, SETD1A, RNF40, DHRS11, ACACA, ENSSSCG00000007733, CCT6A, STX1B, ELOA, ZNF713, HSD3B7, ENSSSCG00000033141, VKORC1* and *KCTD7.*

### Functional analysis and gene enrichment

**Fig. 5:**
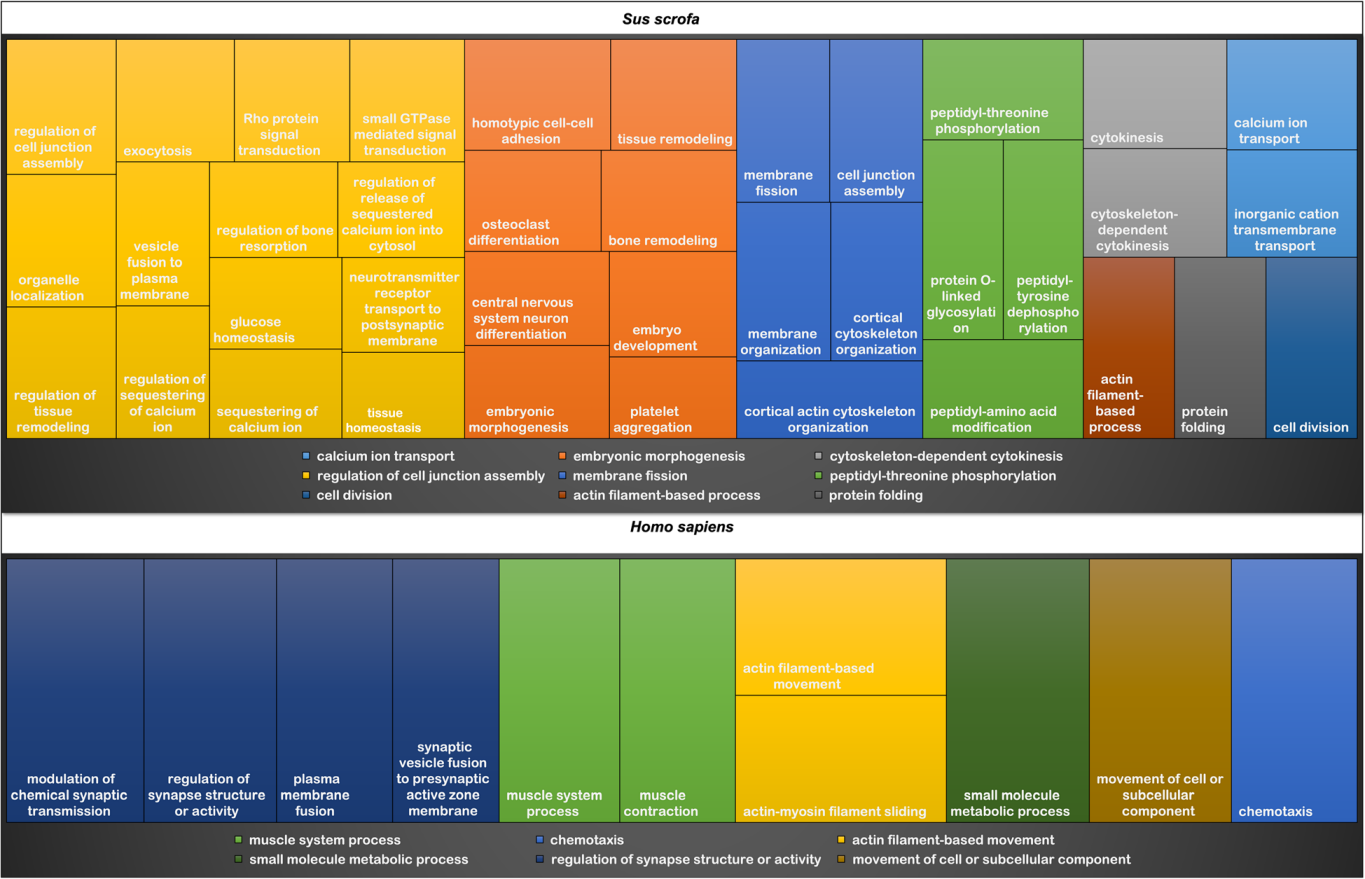
Superclusters of biological processes enriched by genes involved with umbilical hernia found in the exome and transcriptome approaches. Different colors show different superclusters and the size of each box is determined by the uniqueness of the categories

The gene ontology analysis carried out with all 64 genes obtained through the analysis of the exome and transcriptome was performed in the DAVID and Panther databases with pig and human information. Considering the analysis using the pig information, the main enriched BP were grouped in eight superclusters (Fig. 5): regulation of cell junction, embryonic morphogenesis, membrane fission, phosphorylation, calcium transport, protein folder, cell division, cytokinesis and actin metabolism. When the genes were evaluated with human information, besides those found in pig, it was observed that the genes have also been involved in BP of actin filament-movement, muscle processes and chemotaxis (Additional file 1: Table S5, Fig. 5).

**Fig. 6:**
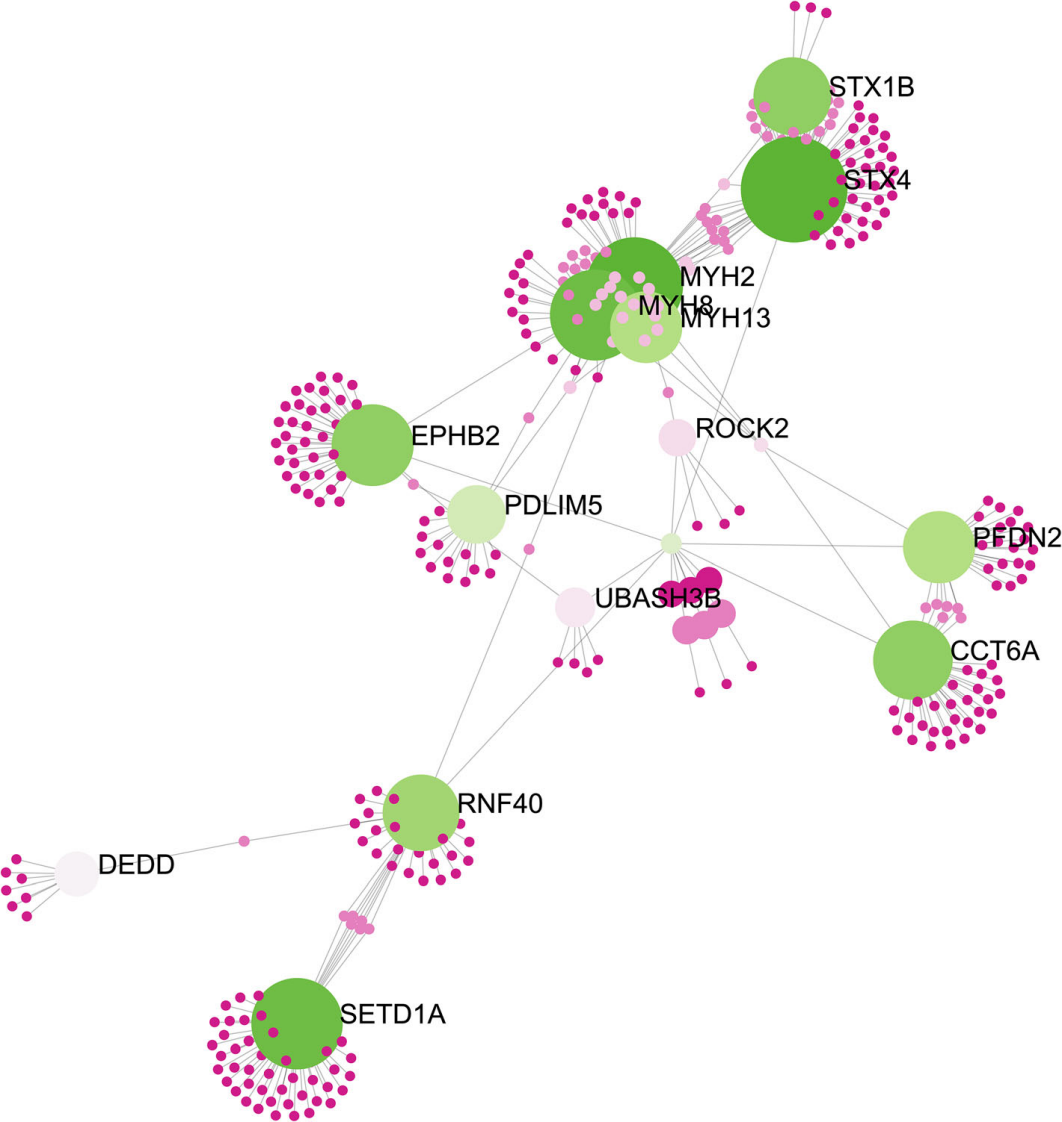
Gene network related to umbilical hernia in pigs constructed the NetworkAnalyst tool using the iMex interactome database. Nodes indicate the number of predicted gene interactions. Strong and large circles contain high number of genes. Green circles are the hub genes

To elucidate the interaction between genes and BPs, the 64 genes obtained through the two methodologies were used to build a network with the NetworkAnalyst tool using the String database (Fig. 6). It is possible to observe some hubs with myosin genes such as *MYH13, MYH2* and *MYH8,* which are mainly involved with the formation of myosin and differentiation of actin and myosin filaments (Fig. 6). When analyzing the exome and transcriptome, several variants were identified located in some genes of the myosin family, responsible for muscle contraction (Additional File 1: Table S2, Fig. 6). Among the main variants, the SNP rs335136145 identified in the *MYO19* gene and located in a missense exon region can be highlighted. In the *MYH13* gene, two SNPs (rs32499668 and rs341831793) were identified in intron and synonymous regions. In the *MYH8* gene, two new variants were identified and in the *MYH2* and *MYH3* genes, two and one new variants were found, respectively (Additional file 1: Table S1, Fig. 6).

**Fig. 7:**
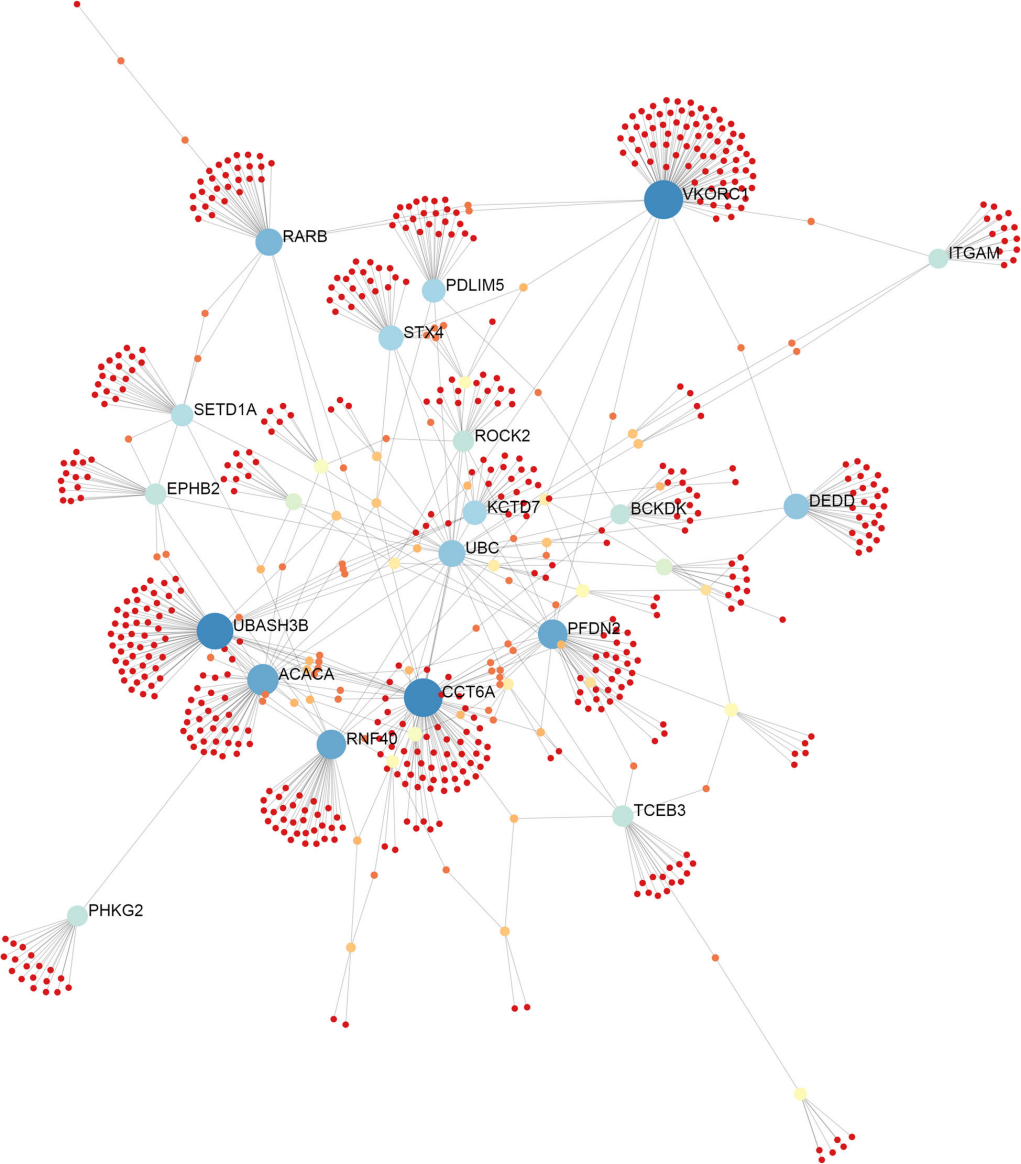
Gene network related to umbilical hernia in pigs constructed in the NetworkAnalyst tool using the String interactome database. Nodes indicate the number of predicted gene interactions. Strong and large circles contain high number of genes. Blue circles are the hub genes

To explore more the results, we also have performed a gene network using IMEx Interactome available in the NetworkAnalyst tool and, beyond the genes already listed, some of them such as *VKORC1, ITGAM* and *CCT6A* could be considered hubs, since they are involved in the expression of several other genes in the network (Fig. 7). Also, a SNP (rs331463738) was identified in the 3 ‘UTR region of the *DHRS11* gene on SSC12, which is responsible for oxidoreductase activity and coenzyme binding. Important genes involved in the carbohydrate metabolic process, such as the alpha-L-fucosidase (*FUCA1*), phosphorylase kinase catalytic subunit gamma 2 (*PHKG2*) and solute carrier family 5 member 2 (*SLC5A2*) genes, have also been found interacting among them (Fig. 7, Additional file 1: Tables S1 and S2). Furthermore, SNPs were also found in *ACACA, BCKDK,* syntaxin 4 (*STX4*) and *STX1B* genes (Fig. 6, Fig. 7), which are involved with amino acid catabolic processes and regulate several other genes in the network.

All five variants selected for Sanger validation were confirmed as shown in the Additional file 1: Fig. S1. The resulting electropherograms showed the point mutation for each chosen gene comparing a normal and an umbilical hernia-affected pig. These results demonstrate the ability of our approach in identifying reliable variants.

## Discussion

The knowledge regarding genes that act in the UH formation is still scarce. Understanding the role and function of the variants in genes is extremely important since they can alter their function and expression and, as a consequence, can directly influence the formation of some anomalies, such as UH [4, 26]. Despite the importance of this problem in pig farming, the knowledge in this field is still limited, although some previous studies using different methodologies pointed out some candidate genes and important biological processes related to umbilical hernia in pigs [13, 27]. To fill this gap, we have generated the first WES of healthy and UH-affected pigs. Moreover, this is the first study integrating the WES and RNA-Seq methodologies to identify putative variants and genes involved with the development of UH in Landrace pigs.

Although the small sample size used was a limitation of our study, we tried to make our experimental design as balanced as possible, using unrelated animals from the same sex and age, originated from the same nucleus farm, which has the same management conditions. To reduce false positive results we were very strict in our variant calling quality control and only selected SNPs with the same genotype within group which differed between case and control groups. Nevertheless, since we could not perform the validation of the polymorphisms in a broader population, further studies should be carried out to test their utility as markers in larger populations.

Several genetic [12, 28, 29] and environmental factors contribute to the formation and development of UH [4, 26]. Some environmental factors like infection of the umbilical cord during birth [2] contribute to the development of hernias. Furthermore, it is known that the weakness of muscle tissue around the umbilical area interferes in the closing of the umbilical canal allowing the intestinal loops to project through the abdominal wall [4, 30].. However, the genetic control of UH is still unknown. A hereditary cause has been suggested by Searcy-Bernal [8], who carried out a progeny test in purebred pigs and showed that the chance of finding a pig with umbilical hernia is different between breeds. Some studies [12, 28, 29] revealed that the development of UH is a polygenic trait, which justifies that just a few candidate genes have already been reported for UH [31]. Therefore, in the current study, polymorphisms and genes related to the formation of UH were identified. The decision for using both WES and RNA-Seq methodologies was to validate the variants found with both approaches to minimize calling spurious sites. Furthermore, using both datasets was possible to identify variants that are present in the DNA and to detect variants beyond protein-coding regions, such as UTR and intronic regions. Moreover, WES also allowed us to identify variants that were present in the DNA but were not expressed in RNA-Seq. A total of 119 variants were identified differing between normal and UH-affected pigs from the exome sequencing (Additional file 1: Table S1), which were located in 58 genes. In the transcriptome, 46 variants were identified (Additional file 1: Table S2) in 27 genes, with 34 of these variants being concordant with the exome, comprising 17 genes common to both approaches. To better clarify the UH etiology, the identification of genes and biological processes involved with this disorder is essential. From the 62 BP found in our study (Fig. 5, Additional file 1: Table S5), cell-matrix adhesion, muscle processes, cell regulation and ion transport can be highlighted. Altogether, our results allowed a close observation of the relationship of the genes with these BP, as further discussed.

### Muscle contraction processes related to UH

It is suggested that the muscle tissue plays an important role in the development of umbilical hernia [29]. In children, it has been reported that the muscles of the umbilical region influence the development of the umbilicus [32]. Xu et al. [33] deduced by histologically examining human fetuses with 8 to 40 weeks of age that muscle contraction probably plays a critical role in closing the umbilical ring after birth, tracing a strong correlation between the umbilicus and the abdominal wall. In our study, several polymorphisms (Additional file 1: Table S1) were identified in genes related to the muscle contraction BP using WES (Fig. 6, Fig. 7). The *MYH13, MYH2, MYH3, MYH8, MYO19* and *ROCK2* genes are responsible for the formation of myosin, actin filaments and protein kinase, skeletal muscle development and muscle contraction [34–38].

Several variants have been identified in the myosin gene family: two polymorphisms were identified in regions of exon (synonymous variant) and intron of the *MYH13* gene (Additional File 1: Table S1). This gene is a fundamental component for the microfilament motor activity and actin-binding [35]. One variant in the upstream region of the *MYH3* gene was identified. *MYH3* function includes nucleotide binding, motor activity and protein binding [39]. In the *MYH2* gene, another member of the myosin family, two new variants located in regions of exons (missense and synonymous variant) and intron were identified (Additional File 1: Table S1). The *MYH2* is responsible for the generation of mechanical force in eukaryotic cells and skeletal muscle contraction [35]. Moreover, in the *MYH8,* a new polymorphism was identified in exon with a missense effect (Additional File 1: Table S1), which was also confirmed with Sanger sequencing. This gene is predominantly expressed in fetal skeletal muscle [39].

In the mammalian genome, myosin is composed by 16 genes, encoding proteins expressed in muscle and non-muscle tissues [35]. In our results, we identified variants in five myosin genes. Xu et al. [33] identified several genes of the myosin family in skeletal muscle in humans and observed that changes in the expression of this family of genes interfere with muscle contraction. Here, the results indicate that the variants found in myosin genes can be strong candidates to trigger UH in pigs, because the musculature, in particular muscular contraction, is extremely important to prevent the passage of the intestinal loops through the umbilical ring causing UH [29]. Furthermore, genes from the myosin family, the 1/3 myosin light chain skeletal muscle isoform (*MYL1)* and myosin light chain 3 (*MYL3)* have been described as candidate genes to the development of scrotal hernia in pigs [35], emphasizing the importance of muscle contraction in the development of hernias. It is interesting to note that although we have several variants in the myosin family, those were not detected in the transcriptome variant analysis neither differentially expressed between normal and affected groups [13]. The non-identification of these variants in the transcriptome could be due to some reasons, such as the small number of reads in the transcriptome, the high number of isoforms, the lack of good quality sequences in those reads or even the low expression profile of these genes at the time of sample collection.

Moreover, a variant was identified in an exomic region (missense) of the *MYO19* gene (Additional File 1: Table S1), which is responsible for ATP binding and actin-binding [33]. Finally, on SSC3, a new deletion in a downstream regulatory region of the *ROCK2* gene was identified (Additional File 1: Table S1). This gene is involved in regulation of smooth muscle contraction, actin cytoskeleton organization, stress fiber and focal adhesion formation [38]. *ROCK2* is a key regulator of the actin cytoskeleton that acts in the formation of the actin/myosin filaments [21]. Human studies indicate that *ROCK2* promotes cancer growth, in addition to degrading MMP2 [40]. In mouse, the lack of this gene can cause cardiac hypertrophy [41]. Therefore, this mutation in the *ROCK2* gene could impair the formation of the actin/myosin filaments, preventing the complete formation of muscle tissue around the navel, leaving this region flaccid and susceptible to the formation of UH.

### Cell-matrix adhesion

The cell adhesion BP is related to the formation of UH since it is responsible for cell connections, cell adhesion, tissue development and maintenance, cell differentiation, migration and communication [40]. Some genes identified in the current study were classified in BPs, such as cell adhesion and regulation of cell junction, for example, the ubiquitin associated and SH3 domain containing B (*UBASH3B*), *RAPGEF5*, *ROCK2* and *EPHB2* (Additional file 1: Tables S1 and S2).

Most of these genes have functions that can be related to the herniation process. The *RAPGEF5* gene is a member of ras family, which acts on cell signaling, recycling and also acting as a ubiquitin ligase [42]. The *UBASH3B* gene can inhibit the endocytosis of the epidermal growth factor receptor (EGFR) and promote the accumulation of activated target receptors, T cells and EGFR on the cell surface [43]. The *EPHB2* is involved in several cellular processes, including cell motility, division and differentiation [44].

In the current study, the *RAPGEF5* presented one new SNP and the *UBASH3B* another SNP (rs345798145), both located in upstream regulatory regions. These genes are involved with increasing levels of epithelial growth factors (EGF) and transforming growth factor (TGF) [45]. Studies have indicated that when there is injury, epithelial cells, macrophages and fibroblasts produce growth factors such as EGF and TGF to heal the injury [46, 47]. Further, in the injury site, there is an increase in the rates of healing and regeneration of the composite fibrous tissue by Fibulin [48, 49]. However, when these growth factors are not balanced, the problem becomes chronic [50]. Probably, those SNPs in the *RAPGEF5* and *UBASH3B* genes could modulate the expression of growth factors, since these variants are located in upstream regulatory regions. Therefore, they may trigger an immune disorder in the umbilical ring tissue, favoring the defense cells to attack the tissue itself, thus, favoring the occurrence of umbilical hernias in pigs.

The *ITGAM* plays a very important role in the cell adhesion process promoting cell binding and in our study we have identified 3 polymorphisms in this gene, including missense mutation. This gene is also involved in several receptor interactions of monocytes, macrophages and granulocytes [46]. Integrin *ITGAM/ITGB2* is also a fibrinogen receptor and regulates the migration of neutrophils [47] while *STX4* acts on the coupling of transport vesicles [48]. Umbilical disorders, including omphalophlebitis, present a significant challenge to the health and well-being of a newborn [39]. Omphalophlebitis is an inflammation or infection of the umbilical region [49], which is the main cause of abscesses [50]. In pig farming, this condition can be caused by mismanagement of cutting or cleaning the umbilical cord [12, 51]. The omphalophlebitis can develop in animals with compromised immune systems and concomitant health problems [52]. Therefore, these *STX4* and *ITGAM* genes might be involved in several processes related to hernia development, such as cell adhesion and also inflammation.

### Genes located in UH QTL regions

Some studies using different approaches have already been developed seeking to identify QTLs for umbilical hernia in pigs [9, 12, 27]. We identified through the exome analysis that the papillin proteoglycan like sulfated glycoprotein (*PAPLN*) gene was located in a QTL region already described in the literature for umbilical hernias in pigs [9].

The *PAPLN* is a component of the extracellular matrix [51], widely studied in humans, and it has been related to liver diseases and growth [52, 53]. It is one of the main glycoproteins in the extracellular matrix [54]. Suppression of Papillin in *Drosophila* has already been associated with embryonic death during embryogenesis due to disorders and abnormalities in muscle formation and Malpighi tubules malformation [53]. Moreover, in humans, the Papillin is known to have an important role in the modulation of metalloproteins during organogenesis, acting directly in the differentiation of the three germ leaflets [52]. This indicates that this gene can interfere in the differentiation of ectoderm, mesoderm and endoderm for the formation of organs. However, there is not much information about the performance of this gene in pigs and this is the first time that this gene has been related to the development of UH.

### Concordant variants and candidate genes from both methodologies

Combining the results from the two methodologies and those from the VEP tool using the SNP database, we evaluated the consequences of the polymorphisms found when they were predicted to affect the production of amino acids. Fourteen variants in the exome and seven variants in the transcriptome were classified with the SIFT score (Table 5). Polymorphisms in the regulatory and coding regions of the genome may be implicated in the development of diseases and congenital problems. Generally, non-synonymous SNPs, such as missense type variants, lead to amino acid changes in protein products, in which they represent approximately half of the known genetic problems responsible for human hereditary diseases [54]. Variants in the *MYH8, MYH2, ENSSSCG00000036685* and *MYO19* genes were identified only in the WES while variants in the *ZNF713, ZNF629, ITGAM*, *SET1DA, RNF40, ORAI3,* and *ELOA* were identified by the two methodologies. The zinc protein family genes identified in our study, such as *ZNF629* and *ZNF713,* are associated with protein-protein interactions, with important role in the transcription and translation regulation [55] that have already been associated with bone and joint malformations, abnormalities of the skin, hair, teeth and nails [56]. Some zinc fingers have already been associated with other congenital diaphragmatic hernia [57]. Furthermore, according to gene network obtained in Stringdb, there are interactions of *ZNF629* and *ZNF713* with some other genes found in our study, such as *CCT6A* and *SUMF2.* However, we emphasize that in pigs there is no study associating these genes to the development of different diseases and disorders, so there is still little information on the role of these genes in the literature, even in humans. Therefore, it is the first time that these genes are associated with the development of umbilical hernia in pigs.

The *RNF40* gene has an important function on histone and gene regulation, being required to active the Hox genes [58]. In mice, its expression is associated with Ubiquitin-protein ligase E3 acting on the degradation of syntaxin 1 [59], encoded by a gene in which polymorphisms were identified. According to Schneider et al. [59], there is a strong relationship between the *RNF40* and *STX1* in cell apoptosis regulation. Therefore, the variants identified in *RNF40* gene (rs1107804156, rs789266896 and a new deletion) (Additional file 1: Table S4) may be influencing the cellular apoptosis of umbilical cells favoring the UH appearance.

The elongin A gene (*ELOA*) encodes a protein expressed in epithelial cells responsible for the elasticity of epithelial tissue [60]. The high expression of this gene in humans is related to the high epithelial cell density [61], promoting epithelial layer formation. In herniated pigs, epithelial tissue resistance related to the development of umbilical hernia was highlighted by Souza et al. [13]. In a previous study of our group, Rodrigues et al. [62] have already described variants potentially associated to UH in *ELOA, ZNF713, ITGAM* and some other genes using RNA-Seq analysis. Now, with this study we were able to find some of the variants in both WES and RNA-Seq, highlighting these genes as candidates for UH in pigs.

The *ORAI3* gene is related to cellular calcium storage activity [63] while the *KCTD7* gene is related to potassium channel tetramerization [64]. Both potassium and calcium channels are fundamental for the normal functioning of cells and the lack of these compounds can cause cell death, which may weakening the tissues due to the lack of cell structure [65].

Some of the variants located in genes were identified with both WES and RNA-Seq approaches. Therefore, this shows that some variants, mainly of the missense type, that alter proteins are strong candidates as factors predisposing the occurrence of UH. These variants have been identified in many genes present in several BP; some of them discussed above are key genes for triggering UH. However, although we have identified functional variants, none of the genes were differentially expressed in our previous publication [13]. This could indicate that some of the variants/genes could have a role in early stages of development. These results show that the variants found in our study are fundamental pieces for understanding the etiology of the UH.

Exome sequencing has been widely used to search for protein-coding genes responsible for causing human disorders. This approach is an alternative to WGS and has been helpful to identify protein-coding mutations related to a diverse number of traits including hernia in pigs. In this study, the probes used for exome selection were designed based on the previous pig genome version, while we have annotated in the latest version of the genome to have more updated results. Thus, it is important to note that these differences in the genome annotation could allow the identification of non-exomic regions and possibly fail to detect some of the new annotated genes. However, we did not consider this an issue, since using both WES and RNA-Seq approaches were interesting to evince and validate some of the DNA variants and to detect variants in non-coding regions that could also be important for regulatory functions. In this way, the use of both strategies complements each other. Moreover, it is interesting to pointed out that unique polymorphisms were found in the RNA-Seq analysis, possibly due to library preparation artifacts, the presence of RNA editing and splicing sites and also because this approach covers other regions than the exome sequencing, such as UTR and intron regions. Furthermore, although we have used a small number of animals, we have been conservative to call variants, which allowed us to identify and also validate polymorphisms in genes that could be considered candidates for the development of UH in pigs. However, further studies are required to validate the association of these groups of polymorphisms and genes in the development of UH in larger populations. Nevertheless, the combination of the two methodologies used greatly improved the reliability of our results, providing the discovery of variants possibly involved with the onset of UH and the paths to understand the umbilical hernia development.

## Conclusions

We have generated the first exome sequencing related to normal and umbilical hernia-affected pigs, which allowed us to identify several variants involved with this disorder. Moreover, comparing these variants with the results from RNA-Seq, it was possible to validate some variants present in the DNA, and to identify those polymorphisms in genes and other regulatory regions that had not been previously related to the development of umbilical hernia in pigs. Muscle contraction and cell-matrix adhesion were the main active biological processes related to the umbilical hernia occurrence. These results contribute to better understand the complex mechanisms involved with the occurrence of UH in pigs and possibly in other mammals, including humans. Some variants found in the genes can be highlighted as strong candidates to the development of UH in pigs and should be further investigated.

## Supplementary Information

**Additional file 1: Table S1.** Variants (SNPs and InDels) identified in the whole-exome and RNA sequencing that differed between normal and umbilical hernia-affected pigs, and their respective genotypes. **Table S2.** Total number of variants (SNPs and InDels) identified in the whole-exome sequencing that differed between normal and umbilical hernia-affected pigs and its annotation and consequence predicted with the VEP tool. **Table S3.** Total number of variants (SNPs and InDels) identified in the transcriptome analysis that differed between normal and umbilical hernia-affected pigs and its annotation and consequence predicted with the VEP tool. **Table S4.** Polymorphisms and genes associated to umbilical hernia in pigs annotated in the exome and transcriptome analyses. **Table S5.** GO biological process enriched in the DAVID (A) and Panther (B) databases based on the candidate genes identified for umbilical hernia through the analyses of the whole exome and RNA sequencing data. **Fig. S1.** Electropherogram of all five variants chosen to be validated with Sanger methodology

## Data Availability

The datasets used or analyzed during the current study are available from the corresponding author on reasonable request. The transcriptome sequences are available in the SRA database with BioProject number PRJNA445856. The polymorphisms were deposited in the EVA database Project number PRJEB48002 with the IDs for RNA-Sequencing: ERZ3677553 and exome sequencing: ERZ3677554.

## Abbreviations

UH: umbilical hernia
BP: biological processes
CEUA: Ethical Committee for Animal Use
VEP: variant effect predictor
QTL: Quantitative Trait Loci
SNP: single nucleotide polymorphism
InDel: insertion and deletion
QC: quality control
GO: Gene Ontology
SIFT: Sorting Intolerant From Tolerant
NGS: Next Generation Sequencing
WES: Whole-Exome Sequencing
